# Genetically supported causality between benign prostate hyperplasia and urinary bladder neoplasms: A mendelian randomization study

**DOI:** 10.3389/fgene.2022.1016696

**Published:** 2022-11-17

**Authors:** Wenzhi Du, Tianyi Wang, Wenxiu Zhang, Yu Xiao, Xinghuan Wang

**Affiliations:** ^1^ Department of Urology, Zhongnan Hospital of Wuhan University, Wuhan University, Wuhan, China; ^2^ Department of Internal Medicine, Zhongnan Hospital of Wuhan University, Wuhan University, Wuhan, China; ^3^ Department of Pediatrics, Maternal and Child Health Care Hospital of Shandong Province, Jinan, China; ^4^ Department of Biological Repositories, Zhongnan Hospital of Wuhan University, Wuhan, China

**Keywords:** mendelian randomization, genome-wide association study, benign prostate hyperplasia, bladder cancer, causal inference

## Abstract

**Background:** Observational studies have suggested a possible association between benign prostate hyperplasia (BPH) and bladder cancer (BLCA). However, these studies are prone to errors and limitations or confounding factors, making them unsuitable for assessing the causal relationship between BPH and BLCA.

**Objective:** Two-sample Mendelian randomization (MR) was performed to determine a possible association between genetically predicted BPH and the risk of BLCA.

**Methods:** A two-sample MR analysis was performed utilizing the Integrative Epidemiology Unit genome-wide association (GWAS) database of the Medical Research Council, United Kingdom A series of control steps, including five primary methods, were performed to identify the most suitable instrumental variables (IVs) for MR analysis. Sensitivity analysis was conducted to avoid statistical errors, including heterogeneity and pleiotropic bias.

**Results:** Genetic variants associated with BPH (P < 5 × 10–8) and BLCA (P < 5 × 10–6) were identified as instrumental variables and assessed using GWAS summary data (BPH, 4,670 cases vs. 458,340 controls; BLCA, 1,279 cases vs. 372,016 controls). BPH exhibited a positive effect on the occurrence of BLCA (inverse variance weighted (IVW), odds ratio (OR) = 1.095, 95% confidence interval (CI) = 1.030–1.165, *p* = 0.003), but there was no causal effect for BLCA on BPH (IVW, OR = 1.092, 95% CI = 0.814–1.465, *p* = 0.554).

**Conclusion:** Genetically predicted BPH was associated with a higher risk of BLCA in all histological subtypes. In contrast, the evidence was not significant to back the causality of genetically induced BLCA on BPH. These findings indicate that BPH plays a key role in developing BLCA in the European population. Further studies are needed to uncover the underlying mechanisms.

## Introduction

Benign prostate hyperplasia (BPH), obstructive disease of the lower urinary system, is more prevalent among men of age more than 50 years, accompanied mainly by lower urinary tract symptoms (LUTS) (Chughtai et al., 2016), subsequently leading to bladder outflow obstruction (BOO), impairing urine emptying, producing residual urine (PVR) (Roehrborn, 2008; Foo, 2019), and reducing the quality of life. Long-term BOO can lead to bladder fibrosis, causing decreased bladder compliance and changes in postganglionic cholinergic fibers (Chertin et al., 2003; Wang et al., 2021a). In the early stage, the symptoms are primarily atypical because of forced urinary muscle function compensation. As the lower urinary tract obstruction gradually aggravates, the clinical symptoms become increasingly apparent, with the main manifestations being frequent urination, urgency, dysuria, increased nocturia, and hematuria (Irwin et al., 2011). Several studies have uncovered genetic determinants or biomarkers of BPH risk to provide essential guidance for novel pharmacotherapies or risk prediction (Sengupta et al., 2008; Zeng et al., 2009; Desaulniers et al., 2017; Hellwege et al., 2019).

Bladder cancer (BLCA) is the most common cancer of the urinary system, ranking 13th in mortality among all malignant tumors worldwide (Mao et al., 2021; Xiong et al., 2021). The number of new cases of BLCA in the US has increased to more than 80,000, with global mortality of 200,000 (Lenis et al., 2020). The development of BLCA is closely associated with genetic abnormalities and epigenetic changes. Tobacco use (Freedman et al., 2011), chemical exposure (Cumberbatch et al., 2018), radiation therapy, and chemotherapy are the leading causes of BLCA. Although both BPH and BLCA are independent diseases with evidently different pathophysiological changes, epidemiology, and risk factors (Chughtai et al., 2016; Lenis et al., 2020), the coexistence of BPH and BLCA has often been observed in many patients (Kang et al., 2007; Tseng, 2013). Nonetheless, there is little evidence to establish a definite causality between the two diseases. According to some studies, the risk of BLCA increases with BPH (Dai et al., 2016; Ham et al., 2021), and patients with a family history of BLCA may be more likely to have BPH (Negri et al., 2005). However, as an unavoidable inherent disadvantage, traditional observational studies have several limitations that weaken the reliability of their findings regarding causal effects (Thiese, 2014). Thus, there is a need to establish the causal connection between the two diseases to understand the etiology of disease processes and identify a novel therapeutic modality.

Mendelian randomization (MR) can estimate the exposure and outcome of a disease by estimating its genetics, especially in cases with insufficient evidence to conduct a randomized controlled trial (Duan et al., 2021). For MR, researchers use instrumental variables (IVs) comprising single-nucleotide polymorphisms (SNPs) (Noordam et al., 2016) to perform causal analysis, making MR more resistant to reverse causality, bias, and confounders compared to traditional observational studies. This method has been widely used in epidemiological studies to show the causal link between conditions and diseases (Wang et al., 2021b). The application of MR should be based on critical assumptions that—despite confounding factors—genetic variants are highly correlated with exposure and associated with the outcome only after exposure (Wang et al., 2022). Instead of relying on genetic variants from individuals, MR estimates the exposure-outcome relationship using a summary of genome-wide association (GWAS) data (Zhao et al., 2021). No MR study has examined the causality between BPH and BLCA. Based on all the above-mentioned information, we hypothesized that while BPH can increase the risk of BLCA, but the latter does not exacerbate the condition of BPH. In this study, we attempted to identify the causal relationships and direction between the two diseases by performing a two-sample bidirectional MR analysis.

## Methods

### Source of data

For this study, data were collected from two large GWAS datasets. When there is a variation between the allele frequencies and exposure distributions of different ethnic groups, the result of population stratification is an association between the outcome and exposure (Larsson et al., 2019). To avoid population stratification bias, we selected genetic variants only from European ancestries, which ensured that the results did not reflect the racial or ethnic composition of the population. The Integrative Epidemiology Unit (IEU) has created the IEU GWAS database at the University of Bristol (Duan et al., 2021). It comprises over 40,000 publicly available GWAS datasets, continuously updated and used for academic research, including studies concerning prostate (Lv et al., 2022) and bladder (Chen et al., 2021). To assess the causal relationship between BPH and BLCA, we downloaded publicly available datasets from IEU summary-level GWAS data (https://gwas.mrcieu.ac.uk/) and performed two-sample bidirectional MR estimates, including BPH (ukb-b-11601) and BLCA (ieu-b-4874). The BPH dataset derives from the IEU consortium of the Medical Research Council (MRC-IEU), including 463,010 individuals (4,670 cases vs. 458,340 controls) and 9,851,867 SNPs of European ancestry. The GWAS dataset for BLCA comprises 373,295 (1,279 cases vs. 372,016 controls) and 9,904,926 SNPs of European ancestry. As mentioned above, we only adopted European ancestry datasets to prevent deviations from population stratification. (Table 1).

**TABLE 1:** Basic information of datasets.

Phenotype	Group ID	Database	Description
Benign prostate hyperplasia (BPH)	ukb-b-11601	UKBiobank	Diagnoses - main ICD10: N40 Hyperplasia of prostate; 9,851,867 SNPs
Bladder cancer (BLCA)	ieu-b-4874	NA	Bladder cancer; 9,904,926 SNPs

Benign prostate hyperplasia, BPH; bladder cancer, BLCA; the effect size of BPH, β, single-nucleotide polymorphism, SNPs.

### Single nucleotide polymorphism selection

A range of quality control standards was established using the above-mentioned datasets, and the specific IVs having solid associations with exposure were identified. First, the identified IVs needed to have a genome-wide significance level. We selected IVs with P < 5 × 10^−8^ from exposures, which can guarantee a significant association of IVs with exposures (BPH or BLCA). In our BLCA data analysis, we detected only two genome-wide significant SNPs (rs146190477 and rs74903734), which may result in weak instruments (Choi et al., 2019). Therefore, we applied a set of genetic instruments to reach a more relaxed threshold (P < 5 × 10^−6^). Then, to meet the above-mentioned basic IV assumptions, we removed linkage disequilibria (LD) —a measure of the association between alleles at related loci (Slatkin, 2008) —to prevent the genetic variation that LD caused. Accordingly, the TwoSampleMR package default parameters were set (physical distance threshold, 10,000 kb; r^2^ < 0.001) for clumping data and shearing the genetic variants that could be possible LD. Then, we used the PhenoSCanner database, which includes a large amount of information about SNPs with possible linkages to diseases and phenotypes (Staley et al., 2016), and found no association of SNPs between BPH and BLCA. Finally, the selected SNPs were not considered biased due to their minor allele frequencies (MAF) (not < 0.01) in the original datasets. From the outcome study, if no proxy variant was identified for a target variant, it was excluded. Similarly, when using the TwoSampleMR package, a palindromic SNP with intermediate allele frequencies must be eliminated to harmonize the outcome and exposure. We evaluated the F statistics to analyze the intensity of the association between exposure and SNPs (set threshold value > 10; formula: *F = β* ^*2*^ */SE* ^*2*^; the effect on the risk of exposure, β; the standard error, SE) (Burgess and Thompson, 2011). One of the SNPs was considered a “weak instrument” (value < 10), and was thus removed from the analysis (Carter et al., 2021). The explained genetic variance was also calculated for each instrument (formula: $VE={\left(SE\times \sqrt{N}\right)}^{2}$ ; variance explained, VE; the number of samples, N).

### Analysis tool

Data were analyzed using R software (4.1.0) and the R packages by MR and MR-Pleiotropy RESidual Sum and Outlier (PRESSO) (Hemani et al., 2018; Verbanck et al., 2018). The code used is openly accessible.

### Mendelian randomization analysis

For statistical analysis, we implemented five approaches, including inverse variance weighted (IVW), MR-Egger, weighted median, simple mode, and weighted mode. First, all selected SNPs were analyzed by the IVW method, which can efficiently find whether directional pleiotropy exists or not. Then, the slope of the weighted linear regression against the horizontal pleiotropy was used in the MR-Egger method to obtain a causal estimate (Burgess and Thompson, 2017). Then, the weighted median estimate was used when 50% of the weight was derived from valid IVs (Bowden et al., 2016a). To further reflect the authenticity of our MR analysis, we adopted the simple mode, weighted modes, and the maximum likelihood ratio test. Lastly, considering that exposure and outcome can interact as both cause and effect, we reversed the role of exposure and outcome in bidirectional MR studies and further established the causal relationship between BPH and BLCA.

### Sensitivity analysis

As a barometer of the MR assumption violations (Bowden et al., 2016b), the degree of heterogeneity of the IVs was evaluated using the IVW and ER-Egger methods. We implemented Cochran’s Q test to quantify the heterogeneity when *p* < 0.05 reflected significant heterogeneity among the causal appraisal for the selected SNPs (Cohen et al., 2015). Moreover, to exclude the possibility that any specific SNP may change the effect proportionately, we performed a leave-one-out plot analysis (Cheng et al., 2017) to determine whether no such distinct SNP exists; this method remarkably decides the confidence and scientificalness of the MR analysis. We employed funnel plots, MR-PRESSO, and MR-Egger to find the potential unbalanced horizontal pleiotropy between the IVs. MR-Egger intercept tool measures the likelihood of a directional pleiotropy of a genetic variant (*p* < 0.05), and the MR-PRESSO test can detect the potential pleiotropy even in a tiny number of loci (Burgess and Thompson, 2017; Hemani et al., 2018). In addition, compared with the MR-Egger regression test, the MR-PRESSO outliner test has higher accuracy (Hemani et al., 2018). In our bidirectional MR analysis, all sensitivity tests mentioned above are two-sided.

## Results

### Benign prostate hyperplasia increases the risk of bladder cancer

From the GWAS dataset for BPH without LD (r^2^ < 0.001), 12 IVs (Table 2 and Supplementary Figure S3) were selected; these were within the 10,000 kb physical distance threshold and achieved genome-wide significance (P < 5 × 10^−8^). To avoid SNPs with palindromic nature and intermediate allele frequencies, we abolished one SNP (rs12131120) with the help of the two-sample MR function of the R package. We conducted the F statistic to ensure that no weak instrument exists, and the results were in the range of 37.1–417.4.

**TABLE 2:** MR effect size of BPH on BLCA with details of selected SNPs.

NO.	SNPs	POS	Chr	β	SE	Effect_allele	Other_allele	F	VE	*P*
1	rs12131120	88,199,778	1	−0.00117	0.000208	A	T	157.274	0.02	2.00E-08
2	rs2556378	60,762,502	2	−0.0017	0.000283	G	T	74.528	0.037	2.10E-09
3	rs380286	1,320,247	5	−0.00186	0.000208	A	G	201.52	0.02	4.90E-19
4	rs1379553	110,979,116	5	−0.00142	0.000256	G	A	96.132	0.03	3.10E-08
6	rs9348716	26,375,658	6	−0.00209	0.000311	A	G	43.201	0.044	1.80E-11
7	rs4266963	22,344,228	10	−0.00149	0.000241	C	T	139.212	0.026	6.90E-10
8	rs12416595	22,409,964	10	0.001364	0.000248	G	C	241.557	0.028	3.90E-08
9	rs10788160	123,033,549	10	0.002116	0.000238	A	G	335.213	0.026	6.10E-19
10	rs12255539	122,614,377	10	0.001769	0.000263	A	G	83.197	0.032	1.80E-11
11	rs3116616	51,162,913	13	0.001568	0.000251	G	C	417.483	0.029	4.20E-10
12	rs11084596	32,104,979	19	−0.00156	0.000216	C	T	37.134	0.021	5.60E-13

Benign prostate hyperplasia, BPH; bladder cancer, BLCA; the effect size of BPH, β; single-nucleotide polymorphism, SNPs; chromosome, Chr; F statistics, evaluate the weak instrument variables, F; variance explained, VE; standard error, SE; *p*-value, *P*

We selected 12 compelling IVs above, we use IVW (*p* = 0.003 < 0.05), MR-Egger (*p* = 0.103 > 0.05), weighted median (*p* = 0.099 > 0.05), simple mode (*p* = 0.497 > 0.05), and weighted mode (*p* = 0.521 > 0.05) methods to analyze the causal effects of BPH and BLCA (Table 3 and Supplementary Figures S1B,C). The IVW method indicated a symbolic association; nonetheless, the results of the five above-mentioned approaches showed no consistency. Therefore, to further establish the credibility of our conclusion, we performed the maximum likelihood method (*p* = 0.003 < 0.05), which complemented the outcomes of the IVW approach and verified the persuasiveness of our study.

**TABLE 3:** BPH & BLCA: Bidirectional MR results.

Methods	The effect size of BPH on BLCA			The effect size of BLCA on BPH		
	OR (95%CI)	Beta	*P*	OR (95%CI)	Beta	*P*
IVW^a^	1.095 (1.030, 1.165)	0.091	0.003	1.092 (0.814, 1.465)	0.088	0.554
MR Egger^a^	1.481 (0.981, 2.235)	0.392	0.103	0.742 (0.140, 3.919)	−0.297	0.732
Weighted median^a^	1.073 (0.986, 1.168)	0.071	0.099	0.927 (0.736, 1.166)	−0.075	0.517
Simple mode^a^	1.053 (0.912, 1.217)	0.052	0.497	0.879 (0.630, 1.227)	−0.128	0.464
Weighted mode^a^	1.054 (0.902, 1.232)	0.053	0.521	0.884 (0.627, 1.249)	−0.122	0.500
Maximum likelihood^a^		0.093	0.003			

Benign prostate hyperplasia, BPH; bladder cancer, BLCA; *p*-value, *P*; inverse variance weighted, IVW.

^a^ No MR-PRESSO, outliers were exposed.

Assessment of the heterogeneity of the selected IVs using the MR-Egger (Q = 5.508; *p* = 0.598 > 0.05) and IVW (Q = 7.613; *p* = 0.472 > 0.05) methods (Table 4) showed that no apparent heterogeneity. Leave-one-out cross-validation was conducted, and no specific SNP was observed to influence the effects disproportionately (Supplementary Figure S1A). The MR-Egger horizontal pleiotropy test suggested the absence of any bias in the results (Intercept = −0.00052, *p* = 0.190 > 0.05). Besides, the funnel plot test revealed the absence of horizontal pleiotropy (Table 4 and Supplementary Figure S1D). Moreover, MR-PRESSO global test and outliner test did not detect any heterogeneity or outliners.

**TABLE 4:** Heterogeneity and horizontal pleiotropy check.

EP	OC	Heterogeneity check 1		Heterogeneity check 2		Horizontal pleiotropy check		
		Q	*P*	Q	P	Intercept	SE	P
BPH	BLCA	7.613744	0.47208	5.508447	0.598165	-0.00052	0.000357	0.190081
BLCA	BPH	34.51226	0.00056	33.85226	0.000382	0.000309	0.000667	0.652321

Benign prostate hyperplasia, BPH; bladder cancer, BLCA; exposure, EP; outcome, OC; Heterogeneity check one was performed by IVW, method; Cochran’s Q value, Q; Heterogeneity check two was performed by MR-Egger method; Horizontal pleiotropy test was performed by MR-Egger method; inverse variance weighted, IVW; standard error, SE.

### Bladder cancer does not increase the risk of benign prostate hyperplasia

We reversed the outcome and exposure location in the bi-direction MR analysis and echoed the above-mentioned causal effect MR analysis approaches of BPH to BLCA. From the GWAS dataset for BLCA with no LD (r^2^ < 0.001), we selected 25 IVs (Table 5 and Supplementary Figure S3) that were within the 10,000 kb physical distance threshold, had acquired genome-wide significance (P < 5 × 10–6) and had no potential pleiotropy as tested by the PhenoScanner tool. These IVs were found in BLCA within the MAF threshold (> 0.01). There were no palindromic SNPs with intermediate allele frequencies. Individual SNPs had F statistics in the range of 36.4 and 401.3.

**TABLE 5:** MR effect size of BLCA on BPH with details of selected SNPs.

NO.	SNPs	POS	Chr	β	SE	Effect_allele	Other_allele	F	VE	*P*
1	rs771137	34,614,520	1	−0.00092	0.0002	A	C	134.596	0.014	3.80E-06
2	rs71439172	25,584,162	2	−0.0009	0.000194	A	G	41.374	0.014	3.70E-06
3	rs6705922	235,693,805	2	−0.0007	0.000147	A	G	237.256	0.008	2.30E-06
4	rs150797373	8,266,421	4	0.003081	0.000611	A	C	345.279	0.13	4.70E-07
5	rs146190477	90,032,521	4	0.002032	0.000344	T	C	103.251	0.044	3.30E-09
6	rs2736103	1,300,401	5	0.000657	0.000138	C	T	87.213	0.0071	0.000002
7	rs11744794	123,846,328	5	0.000731	0.000147	A	T	190.113	0.008	6.60E-07
8	rs7722450	31,077,122	5	0.001699	0.000368	A	G	64.402	0.05	3.90E-06
9	rs146820664	91,417,975	5	0.002733	0.000554	G	C	157.865	0.11	8.20E-07
10	rs62408223	90,949,196	6	0.000859	0.00018	G	A	117.113	0.012	1.90E-06
11	rs392450	60,024,449	8	0.001234	0.000247	A	G	273.015	0.022	5.60E-07
12	rs10094872	128,719,884	8	0.000706	0.000141	T	A	36.485	0.0074	5.90E-07
13	rs146462590	125,571,782	8	0.003215	0.000675	G	T	355.113	0.17	1.90E-06
14	rs7038689	1,709,649	9	−0.00063	0.000136	C	A	174.201	0.0069	3.20E-06
15	rs11793757	133,257,554	9	0.000981	0.00021	A	G	61.33	0.016	3.10E-06
16	rs184321190	17019503	10	0.002795	0.000599	C	T	383.211	0.13	3.10E-06
17	rs74131143	51,058,643	10	0.001644	0.000351	A	G	72.654	0.045	2.80E-06
18	rs71227149	90,852	12	0.002864	0.000597	A	G	131.342	0.13	1.60E-06
19	rs11158808	69,933,892	14	−0.00102	0.000198	T	C	191.44	0.014	3.00E-07
20	rs147813917	59,246,079	15	0.002756	0.000553	A	G	400.135	0.11	6.20E-07
21	rs3744753	907,907	17	0.001666	0.000334	A	G	91.357	0.041	6.30E-07
22	rs35515294	10,358,090	20	0.002107	0.000426	T	C	62.219	0.067	7.40E-07
23	rs6078008	10,995,830	20	0.000687	0.000149	A	T	150.013	0.0082	3.70E-06
24	rs56297045	39,350,684	22	0.000732	0.000156	A	G	401.312	0.009	2.50E-06
25	rs74903734	25,799,984	22	0.003273	0.000589	G	A	217.001	0.12	2.70E-08

Benign prostate hyperplasia, BPH; bladder cancer, BLCA; the effect size of BLCA, β; single-nucleotide polymorphism, SNPs; chromosome, Chr; F statistics, evaluate the weak instrument variables, F; variance explained, VE; standard error, SE; *p*-value, *P*

We repeated IVW (*p* = 0.554 > 0.05), MR-Egger (*p* = 0.732 > 0.05), weighted median (*p* = 0.517 > 0.05), simple mode (*p* = 0.464 > 0.05), weighted mode (*p* = 0.500 > 0.05), and found no persuasive clue of BLCA increasing the risk of BPH (Table 3 and Supplementary Figures S2B–C).

Subsequently, although the heterogeneity was detected in the selected SNPs through the IVW (Q = 34.512, *p* = 0.0005 < 0.05) and MR-Egger (Q = 33.852, *p* = 0.0003 < 0.05) estimation (Table 4), the leave-one-out cross-validation revealed no SNPs that disproportionally influence the effects (Supplementary Figure S2A); besides, the MR-Egger horizontal pleiotropy test (*p* = 0.652 > 0.05) and funnel plot test showed no bias in the results (Table 4 and Supplementary Figure S2D). Furthermore, the MR-PRESSO test also showed no heterogeneity or outliners. In summary, we have reached a stable and reliable conclusion.

## Discussion

Males suffering from LUTS often have BPH, characterized by proliferation features in the central zone of epithelial components and stroma of the prostate (Jahan et al., 2021). The overall annual incidence of LUTS induced by BPH is estimated to be 15/1,000 person-years (95% CI = 14.8–16.1). In individuals aged 45–49 years, the incidence is 3/1,000 person-years, and in those aged 75–79 years, the incidence increases to 38/1,000 person-years (Naderi et al., 2004). More than 30% of patients with severe LUTS need medical or surgical care (Madersbacher et al., 2019). Long-term BOO can cause pathophysiological changes in the bladder. Many experts speculate a possible causal relationship between BPH and BLCA, a highly malignant disease with high recurrence and progression rates. BLCA has a low 5-year cancer-specific survival rate (Yang et al., 2018), causing 550,000 cases worldwide and 17,000 deaths in the US alone annually (Siegel et al., 2019; Richters et al., 2020).

Nonetheless, the published evidence showed no consistency in the causality of BPH and BLCA. According to a few studies, BPH may increase the likelihood of developing BLCA (Fang et al., 2018; Lenis et al., 2020) and is a crucial risk factor for BLCA, especially for patients with type 2 diabetes (Tseng, 2013). In one meta-analysis that systematically reviewed 16 case-control studies and 10 cohort studies, BPH was associated with a higher incidence of BLCA (18). To reduce prostate volume and lower the recurrence rate of BLCA, some experts used 5-α reductase inhibitors or other androgen deprivation therapies (Shiota et al., 2017; Zhu et al., 2021). Besides, simultaneous surgical treatment to cure BPH and BLCA is safe in patients having both diseases; the process adds no greater risk of BLCA recurrence or metastasis rates, and patients enjoy an improved quality of life after the procedure (Zhou et al., 2020; Wang et al., 2021c). However, some researchers found that BPH has no positive effect on the occurrence of BLCA. In a study on a cohort of almost 80,000 Swedish men with BPH, no association was found between BPH and BLCA, except for those patients who underwent transurethral prostate resection (Kang et al., 2007). Some researchers have also found that transurethral resection of the prostate cannot lower the incidence of bladder cancer (Lin et al., 2018). Nonetheless, these conventional observational studies showed the influence of BPH on BLCA and remained vulnerable to the threat of potential confounders, such as smoking, occupation, and heredity, that are unavoidable in crowd screening. Besides, researchers may find chance results in multiple comparisons and subgroup analyses in a cohort study. As these two common diseases afflict middle-aged and older men, it is crucial to understand the causal relationship between them for better clinical management.

Genetic information is always transmitted unidirectionally from parent to offspring, limiting the impact of reverse causation (Swerdlow et al., 2016). At this point, traditional epidemiological studies are equally unavoidable. To further clarify the inconsistencies and provide a valuable guideline for clinical management, we performed the first two-sample and bidirectional MR analysis to assess the causal direction between BPH and BLCA. To establish our hypothesis, we performed an array of quality control approaches and MR analytical methods and tested heterogeneity; any potential pleiotropy (P > 0.05) was not detected in this MR-based bidirectional causality study. The results demonstrate a causal effect between BPH and BLCA, wherein BPH can increase the risk of BLCA, but the opposite was not observed. According to the findings of this study, we suggest some recommendations for patients with BPH or BLCA. First, patients, especially those with long-term BPH and severe LUTS, need to be examined timely and periodically to prevent the occurrence of BLCA and acquire a better living status. Second, patients who suffer from both BPH and BLCA are suggested to adopt early intervention to avert the development of BPH and slow down the malignant process of BLCA. Third, patients with BLCA but not BPH need to be attentive to any prostate condition during diagnosis and treatment to prevent the aggravation of BLCA due to the later occurrence of BPH. Finally, patients with BLCA experiencing urinary tract irritation should seek medical attention to distinguish whether the source of this symptom comes from BLCA alone or combined BPH.

Our research findings are consistent with the conclusions of previous studies. There are many mechanisms by which BPH leads to BLCA. The ratio of acid phosphatase to *β*-glucuronidase in the urine of patients with prostate cancer was only 10% effective in those having BPH (Muntzing and Nilsson, 1972). BPH causes lower urinary tract obstruction, poor urination, urinary retention, and increased *β*-glucuronidase activity in urine, which decomposes *β*-naphthylamine, 4-aminobiphenyl, and benzidine, that do not easily and directly cause carcinogenesis by being converted into 2-amino-1 naphthol that is a carcinogen, thus significantly prolonging the contact time between some carcinogens and bladder mucosa (Melicow et al., 1961). Matsumoto et al. performed an animal study and observed that BOO prolonged exposure to carcinogens and caused a higher risk of BLCA (Matsumoto et al., 2009). Besides, long-term urinary retention can breed bacteria and increase the risk of cystitis and urinary tract infections, especially for patients with diabetes mellitus (Selius and Subedi, 2008; Odabasi and Mert, 2020). With the deposition of crystalline materials and chemical substances (Coe et al., 2016; Barghouthy et al., 2021), bladder stones are easily formed, consequently aggravating bladder inflammation (Ma et al., 2016; Cicione et al., 2018). These complications induce the proliferation or metaplasia of bladder transitional epithelial cells, causing the formation of transitional cell carcinoma and increasing the chance of recurrence. In one review on BLCA diagnosis and management, researchers reported that the bladder diverticulum has a higher risk of developing BLCA, which might be caused by prolonged exposure of the bladder mucosa to carcinogenic substances due to urinary stasis (Walker et al., 2014). Diluting urinary metabolites by taking more water, removing BOO, and increasing the frequency of urination is associated with a lower incidence of BLCA (Zhou et al., 2014). Chronic kidney disease (CKD) is significantly associated with BPH; patients with CKD have a higher risk of developing BLCA than either patients with BPH or CKD only (Chung et al., 2007; Chen et al., 2008). The potential mechanism may be that CKD tends to be accompanied by obstructive urinary tract disease (Rassweiler et al., 2006). In addition, androgens have a high affinity to prostate tissue through the androgen receptors (AR), which can significantly influence the physiological state of prostate tissues and facilitate BPH development (Heinlein and Chang, 2004; Tong and Zhou, 2020), and trigger the occurrence of various bladder carcinomas (Wang et al., 2020a; Martinez-Rojo et al., 2021). Patients with BLCA who received androgen suppression medicine were more likely to survive than those who did not (Wang et al., 2020b; Wu et al., 2021). Both female and male BLCA cells are highly dependent on AR (Sottnik et al., 2021). AR expression or dihydrotestosterone (DHT) treatment can decrease the forkhead box O1 (FOXO1) expression and cause FOXO1 phosphorylation through the protein kinase B signaling pathway, which works as a tumor suppressor to BLCA cells metastasis, migration, invasion, and proliferation (Ide et al., 2020). The AR-FOXO1-AKT axis might be an essential pathway to the development of BLCA.

Our study had several strengths which are listed as follows. First, this is the first MR study to systematically determine the causal relationship between BPH and the risk of developing BLCA in the European population. This study revealed that the occurrence of BPH caused by specific genetic variants could be a potential cause of BLCA and indicates that there exists a unidirectional genetic link between BPH and BLCA, compensating for the shortcomings of previous studies. Second, our two-sample MR analyses are consistent with the conclusions of previous observational studies, making the conclusions more reliable and trustworthy. However, a few limitations of our study warrant our attention. First, the lack of information regarding the degree of BPH and the BLCA stage prevented a subgroup analysis. Second, although the study supported a link between BPH and BLCA, the sample was only derived from the European population, which is not large enough to generalize various ancestries. Third, in correlation analysis with BPH, we could not exclude the sample of females in the BLCA group; this is caused by the limitations of the GWAS database and may cause statistical bias. Nevertheless, although such a potential effect exists, it is relatively small and unlikely to significantly influence our conclusion. Fourth, overlapping participants should not be involved in the exposure and outcome studies of two-sample MR analysis. In this study, we could not assess the extent of overlap between the two samples. However, the bias due to the overlap between the two samples can be minimized using more powerful tools estimated by the F statistic (much more significant than 10) (Pierce and Burgess, 2013; Wu et al., 2020). Fifth, we extracted data from the largest available GWAS of BLCA, and a few genome-wide significant SNPs were identified, which may have led to weak genetic instruments. Therefore, we applied statistical criteria to meet a more relaxed threshold to include more SNPs as instruments; this method has been performed in many MR studies to solve the problem of insufficient genome-wide significant SNPs (Gage et al., 2017; Hartwig et al., 2017). Last, we found positive results only in IVW but not through other MR statistical methods; nonetheless, the conclusion is still reliable because IVW is the most used, primary, and persuasive MR analysis method (Bowden et al., 2016b). This method combined the Wald ratios of exposure and outcome and provides the most accurate estimation for all selected SNPs (Burgess et al., 2013; Emdin et al., 2017). Meanwhile, we also performed a maximum likelihood ratio analysis, whose outcome was consistent with the IVW result.

## Conclusion

This MR study demonstrated that BPH increased the risk of BLCA, but BLCA did not influence the overall risk of BPH. More studies are needed to explore the underlying biochemical mechanism and find potential therapeutic targets for the prevention of these diseases.

## Data Availability

The original contributions presented in the study are included in the article/Supplementary Material, further inquiries can be directed to the corresponding authors.

## Abbreviations

AR, androgen receptors; BLCA, bladder cancer; BOO, obstruction of bladder outflow; BPH, benign prostate hyperplasia; CI, confidence interval; CKD, chronic kidney disease; DHT, dihydrotestosterone; FOXO1, forkhead box O1; GWAS, genome-wide association study; IVW, inverse variance weighted; IVs, instrumental variables; LD, linkage disequilibria; LUTS, lower urinary tract symptoms; MR, mendelian randomization; MAF, minor allele frequencies; OR, odds ratio; PVR, producing residual urine; PV, prostate volume; SNPs, single nucleotide polymorphisms; VE, variance explained.
