# Supplementary material for: Genetically supported causality between benign prostate hyperplasia and urinary bladder neoplasms: A mendelian randomization study
Source: Front Genet. 2022 Nov 17;13:1016696. doi: 10.3389/fgene.2022.1016696 (PMC9713637; doi:10.3389/fgene.2022.1016696)
Supplement: Supplementary file 1 [file DataSheet1.docx]

**Supplementary Materials**

**

**

**Supplementary Figure S1. Benign prostate hyperplasia increases the risk of bladder cancer.**

**A.** Leave-one-out sensitivity analysis for BPH (ukb-b-11601) on BLCA (ieu-b-4874). The red straight line represents all β values after leave-one-out. **B.** The MR effects size for BPH (ukb-b-11601) on BLCA development (ieu-b-4874) were studied in forest plots. The results indicated that BPH could strengthen the hazard of BLCA. **C.** The scatter plot shows the causal effects from BPH (ukb-b-11601) to BLCA (ieu-b-4874). The color of lines represents the different methods' causal effects and is expressly noted on the top; benign prostate hyperplasia, BPH; bladder cancer, BLCA. **D.** The funnel plot detects the outliers.





**Supplementary Figure S2. Bladder cancer does not increase the risk of benign prostate hyperplasia. A.** Leave-one-out sensitivity analysis for BLCA (ieu-b-4874) on BPH (ukb-b-11601). The red straight line represents all β values after leave-one-out. **B.** The MR effects size for BLCA (ieu-b-4874) on BPH development (ukb-b-11601) were studied in forest plots. The results indicated that BLCA could not strengthen the danger of BPH. **C.** The scatter plot shows the causal effects from BLCA (ieu-b-4874) to BPH (ukb-b-11601). The color of lines represents the different methods' causal effects and is expressly noted on the top; benign prostate hyperplasia, BPH; bladder cancer, BLCA. **D.** The funnel plot detects the outliers.


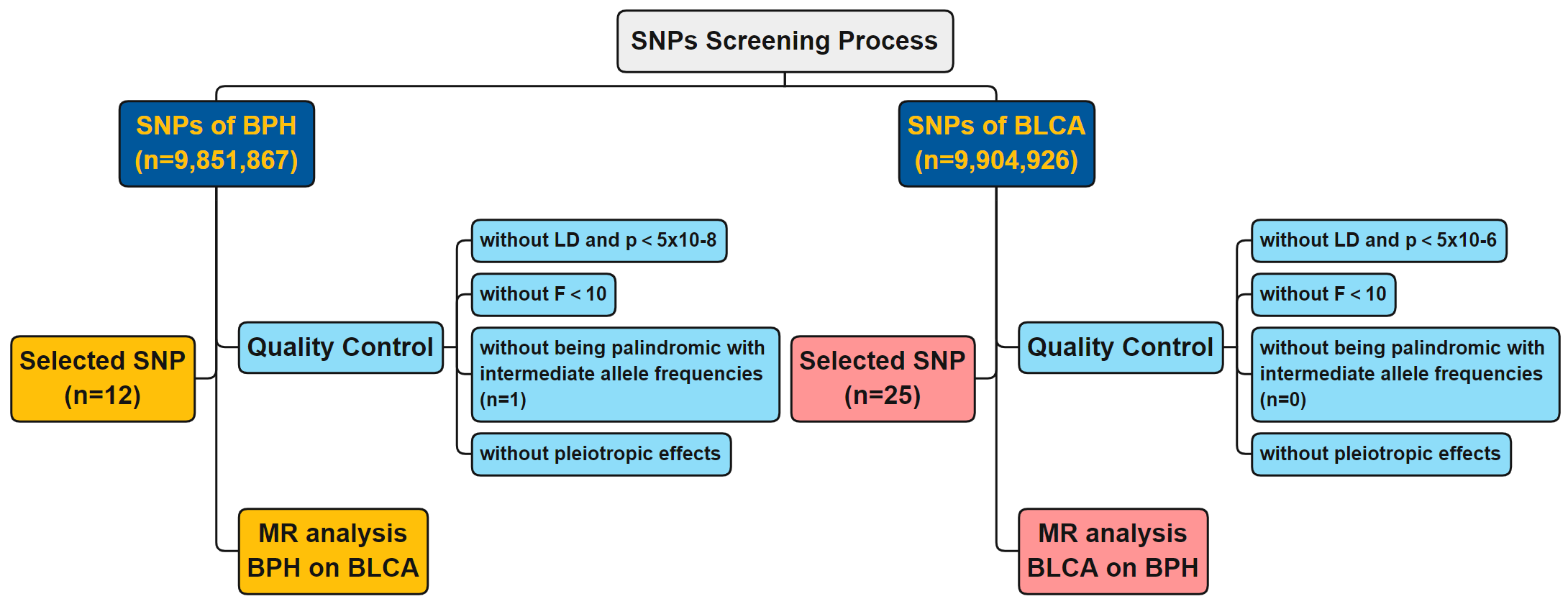


**Supplementary Figure S3. The screening process of SNPs of BPH and BLCA.** Benign prostate hyperplasia, BPH; bladder cancer, BLCA. Mendelian randomization, MR; linkage disequilibrium, LD; single nucleotide polymorphisms, SNPs; p-value, *P*


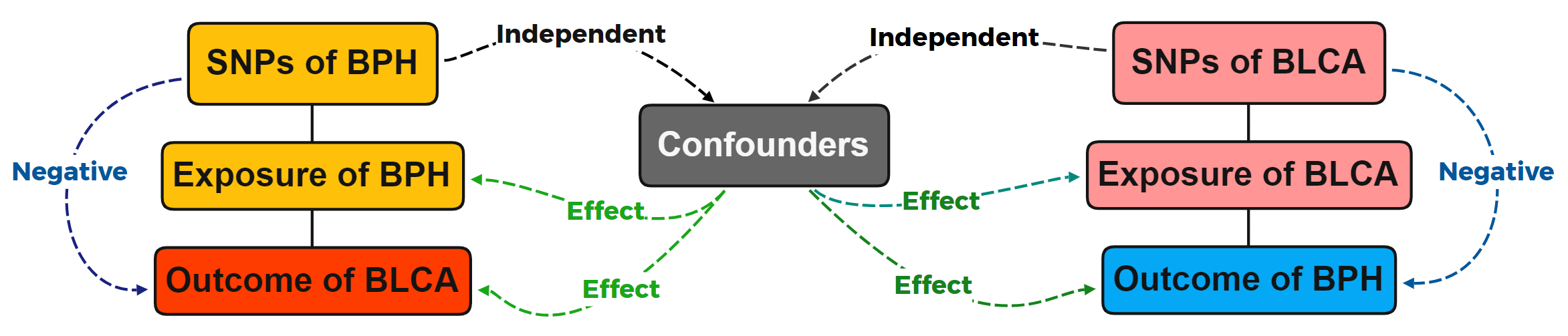
 **Supplementary Figure S4. Schematics for bidirectional MR analysis of BPH and BLCA.** All selected SNPs achieve three basic assumptions. benign prostate hyperplasia, BPH; bladder cancer, BLCA; single nucleotide polymorphisms, SNPs
